# Understanding the Clinical Relationship Between Diabetic Retinopathy, Nephropathy, and Neuropathy: A Comprehensive Review

**DOI:** 10.7759/cureus.56674

**Published:** 2024-03-21

**Authors:** Aditi Kulkarni, Archana R Thool, Sachin Daigavane

**Affiliations:** 1 Ophthalmology, Jawaharlal Nehru Medical College, Datta Meghe Institute of Higher Education and Research, Wardha, IND

**Keywords:** pathophysiological mechanisms, integrated care, diabetic neuropathy, diabetic nephropathy, diabetic retinopathy, diabetic complications

## Abstract

Diabetic retinopathy, nephropathy, and neuropathy are significant microvascular complications of diabetes mellitus, contributing to substantial morbidity and mortality worldwide. This comprehensive review examines the clinical relationship between these complications, focusing on shared pathophysiological mechanisms, bidirectional relationships, and implications for patient management. The review highlights the importance of understanding the interconnected nature of diabetic complications and adopting a holistic approach to diabetes care. Insights gleaned from this review underscore the necessity for early detection, timely intervention, and integrated care models involving collaboration among healthcare professionals. Furthermore, the review emphasizes the need for continued research to elucidate underlying mechanisms, identify novel therapeutic targets, and assess the efficacy of integrated care strategies in improving patient outcomes. By fostering interdisciplinary collaboration and knowledge exchange, future research endeavors hold the potential to advance our understanding and management of diabetic complications, ultimately enhancing patient care and quality of life.

## Introduction and background

Diabetes mellitus is a chronic metabolic disorder characterized by elevated blood glucose levels resulting from insufficient insulin production or the body's inability to utilize insulin effectively. It is a significant global health concern with increasing prevalence rates worldwide. Diabetes can lead to various complications affecting multiple organ systems, including the eyes, kidneys, and nerves [1]. Diabetes-related complications, such as retinopathy, nephropathy, and neuropathy, pose significant risks to patients' health and quality of life (QoL). Understanding the complex interplay between these complications is crucial for healthcare providers to provide comprehensive care and prevent long-term adverse outcomes. The relationship between diabetic retinopathy (DR), nephropathy, and neuropathy often reflects systemic microvascular and macrovascular dysfunction, highlighting the need for a holistic approach to diabetes management [2].

The purpose of this review is to comprehensively examine the clinical relationship between DR, nephropathy, and neuropathy. This review aims to elucidate the underlying mechanisms linking these complications, explore common risk factors, discuss diagnostic and management strategies, and highlight the importance of integrated care approaches by synthesizing current evidence and insights from research and clinical practice. Ultimately, this review seeks to inform healthcare professionals about the interconnected nature of diabetic complications and improve patient outcomes through enhanced understanding and management.

## Review

Diabetic retinopathy

Definition and Epidemiology

DR is a complication of diabetes that impacts the eyes by causing damage to the blood vessels in the retina. It is a primary cause of vision impairment among adults aged 20-74 and ranks the fifth most prevalent cause of preventable blindness globally [3]. In the United States, approximately 9.6 million individuals were affected by DR in 2021, with 1.84 million cases classified as vision-threatening [4]. The prevalence of DR is notably elevated among individuals aged 65-79, with a rate of 28.4%, and it demonstrates a higher occurrence in males compared to females [4]. Non-Hispanic blacks exhibited the highest prevalence rates of DR (3.26%) and vision-threatening DR (1.11%) [4]. DR represents a microvascular complication of diabetes and has the potential to contribute to the onset of other diabetes-related complications, such as peripheral neuropathy and cardiovascular events [3].

Pathogenesis

**Figure 1:**
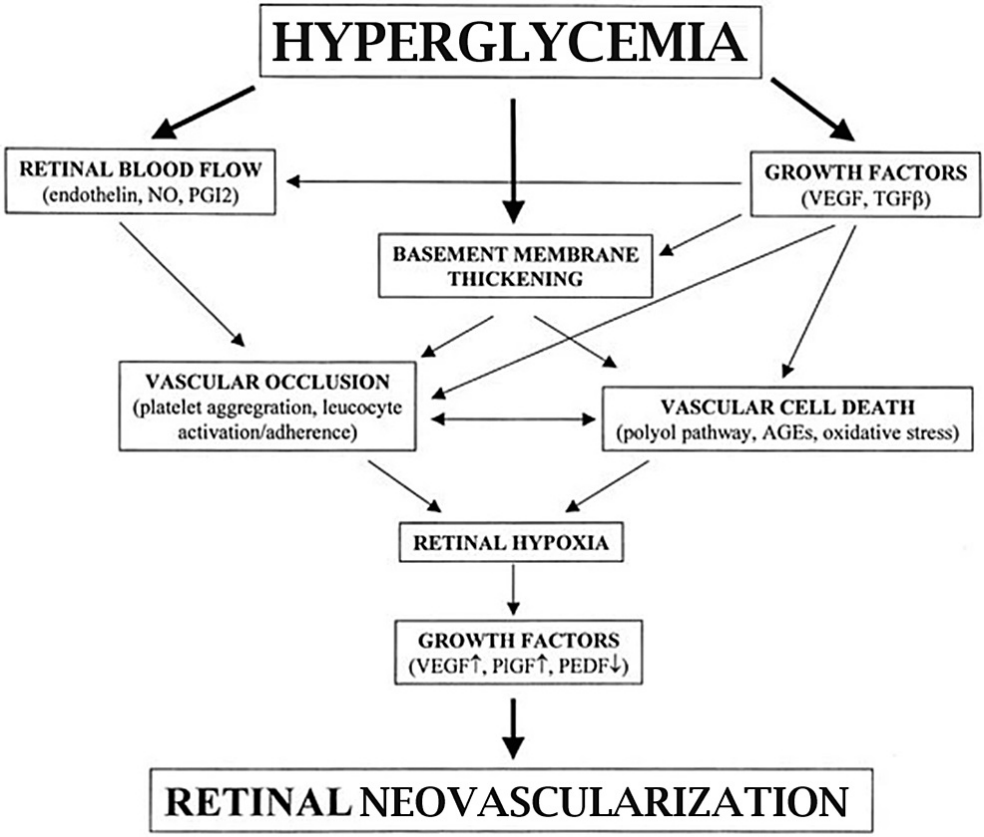
Pathogenesis of diabetic retinopathy Open access journal under a CC-BY license contributed by Dr. M Boulton AGEs, advanced glycation end products; NO, nitric oxide; PEDF, pigment epithelium-derived factor; PIGF, placenta growth factor; PGI2, prostacyclin; TGFβ, transforming growth factor beta; VEGF, vascular endothelial growth factor

The pathogenesis of DR encompasses intricate mechanisms associated with the microvascular complications of diabetes. Hyperglycemia is central in impairing retinal capillaries by fostering the formation of advanced glycation end products (AGEs), thereby instigating endothelial damage and the emergence of microaneurysms (MAs) [5,6]. Concurrently, inflammatory cytokines undergo upregulation in diabetes, instigating chronic inflammation and heightened vascular permeability, thereby contributing to diabetic macular edema (DME) [5,6]. As the condition progresses, sustained ischemia prompts the release of pro-angiogenic factors like vascular endothelial growth factor (VEGF), which fosters neovascularization and holds the potential to precipitate severe complications, such as vitreous hemorrhage or tractional retinal detachment in proliferative DR (PDR) [5,6]. The pathophysiology entails a cascade of events, including capillary occlusion, retinal fluid leakage, and aberrant blood vessel proliferation, all influenced by hyperglycemia, genetic predisposition, hypertension, dyslipidemia, and chronic inflammation [5,6]. Effective management strategies encompass regulating blood sugar levels, serum lipid concentrations, and blood pressure alongside interventions like laser surgery, injections, and eye surgery tailored to the severity of the condition [5,6]. Pathogenesis of DR are shown in Figure 1.

Clinical Manifestations

The clinical manifestations of DR can manifest differently depending on the stage of the disease. During the initial stages, referred to as non-proliferative DR (NPDR), the blood vessels within the retina weaken, leading to the leakage of fluid and blood into the retinal tissue. This leakage can induce symptoms such as blurry vision, dark strings or spots floating in the field of vision (floaters), and gradual vision loss as the condition progresses [7,8]. As the disease advances to PDR, abnormal new blood vessels begin to proliferate on the surface of the retina. These vessels are fragile and prone to breaking, which can result in bleeding into the gel-like substance within the eye known as the vitreous. Such hemorrhages contribute to severe vision impairment [7]. Symptoms associated with DR may include vision fluctuations and the perception of dark or empty areas in the visual field and, if left untreated, may progress to blindness [7]. It is imperative to undergo regular eye examinations, maintain optimal blood sugar levels, and seek early intervention to mitigate the risk of severe vision loss associated with DR [7].

Diagnosis

Diagnosing DR entails a comprehensive dilated eye examination, during which eye specialists administer drops to dilate the pupils, facilitating a thorough examination of the eyes' internal structures [4]. This examination identifies abnormalities in the eyes' interior and exterior components. Furthermore, diagnostic techniques, such as fluorescein angiography and optical coherence tomography (OCT), are employed to evaluate blood vessel irregularities and retinal thickness [4]. Since DR may remain asymptomatic during its initial stages, early detection through routine eye examinations is imperative, underscoring the importance of regular monitoring for timely intervention [7]. Treatment options for DR vary depending on the severity of the condition and may include laser surgery, intravitreal injections, or vitrectomy to manage the disease [9] effectively. Integral to managing DR is the proper control of diabetes, encompassing the regulation of blood sugar levels and blood pressure, which plays a pivotal role in preventing severe vision loss associated with the condition [5].

Management and Treatment

**Figure 2:**
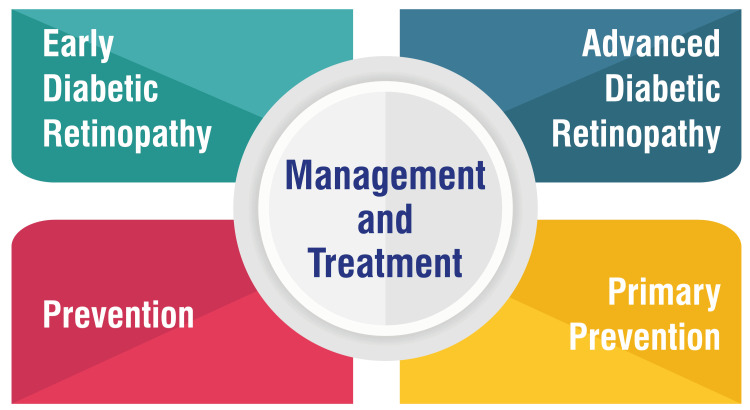
Management and treatment This figure is self-created by the corresponding author.

In the early stages of DR, mild or moderate NPDR may not necessitate immediate treatment. However, vigilant monitoring remains essential to ascertain the appropriate timing for intervention. Achieving and maintaining good blood sugar control is paramount in decelerating or halting the progression of DR [9]. For cases of advanced DR, such as PDR or macular edema, prompt treatment is imperative. Treatment modalities may involve administering medications directly into the eye, such as VEGF inhibitors, to impede the formation of new blood vessels and mitigate fluid accumulation. Additionally, laser treatment and surgical interventions may be recommended, tailored to address specific retinal complications [10]. Efforts geared toward prevention are integral to managing DR. Effective diabetes management through medication adherence, dietary modifications, regular exercise, and meticulous control of blood pressure can serve to either prevent or attenuate the progression of DR. Routine eye examinations are pivotal in enabling early detection and intervention, thereby averting the risk of vision loss [11]. In primary prevention, optimal glycemic control emerges as a cornerstone in mitigating the risk of microvascular complications associated with diabetes. Extensive studies have underscored the pivotal role of maintaining stable glycemic levels in reducing the likelihood of DR progression and subsequent visual impairment [12]. Management and treatment are shown in Figure 2.

Impact on Vision and Quality of Life

Diabetic retinopathy profoundly affects patients' quality of life (QoL) primarily due to vision impairment. Utilising the National Eye Institute Visual Functioning Questionnaire (NEI-VFQ-25), studies have elucidated that diabetic retinopathy, particularly in its advanced stages, is linked with limitations in reading abilities and emotional well-being [13,14]. The severity of diabetic retinopathy correlates with a decline in QoL, impacting various aspects such as independence, mobility, engagement in leisure activities, and self-care. Individuals afflicted with proliferative diabetic retinopathy (PDR) or severe non-proliferative diabetic retinopathy (NPDR) encounter compromised QoL about vision-related functions, consequently encountering hurdles in daily activities and social interactions [15,16]. Despite these impairments in vision-related QoL, certain studies suggest that in populations with good visual acuity, diabetic retinopathy may not adversely affect sleep quality or mood [17]. It is imperative for healthcare providers to regularly assess QoL, utilising tools such as the NEI-VFQ-25 questionnaire to comprehend and address the comprehensive impact of diabetic retinopathy on patients' lives.

Diabetic nephropathy

Definition and Prevalence

Diabetic kidney disease (DKD) stands as a prevalent complication of diabetes, impacting approximately 30%-50% of individuals with type 2 diabetes at any given time, with over 90% of cases in the US attributed to type 2 diabetes. DKD is characterized by heightened urine albumin excretion or diminished glomerular filtration rate (GFR). It represents a substantial contributor to end-stage kidney disease (ESKD), demonstrating a higher prevalence among specific ethnic groups, such as African Americans, Asian Americans, and Native Americans. Annually, the incidence of DKD as a cause of ESKD exhibits an upward trend, with notable racial and global disparities in its epidemiology [18-20]. The prevalence of DKD exhibits variability across studies, spanning from 7% to 94% among all individuals diagnosed with diabetic nephropathy (DN). Particularly noteworthy is the elevated prevalence of DN among specific ethnic demographics, which can precipitate end-stage renal disease. Effectively managing DKD entails the regulation of diabetes and hypertension, with lifestyle modifications assuming a pivotal role. Consistent monitoring and timely intervention are indispensable components in DKD management, aimed at mitigating progression to advanced stages necessitating kidney transplant or dialysis [21,22].

Pathogenesis

The pathogenesis of DN entails a multifaceted interplay of factors, encompassing alterations in glomerular function, hormonal influences, and protein glycosylation. Elevated blood glucose levels induce the glycosylation of glomerular proteins, fostering mesangial cell proliferation, matrix expansion, and vascular endothelial impairment. Disease progression is marked by mesangial expansion, thickening of the glomerular basement membrane, and the emergence of characteristic lesions such as Kimmelstiel-Wilson nodules. Furthermore, renal vasodilation, heightened GFR, and elevated blood pressure are hallmark features of DN [23,24]. While precise etiology remains incompletely understood, the pathogenesis is implicated in hyperglycemia-induced renal injury, advanced glycation products, and cytokine activation. DN represents a primary cause of chronic kidney disease (CKD) in Western societies and a significant complication of diabetes, impacting up to 50% of individuals with longstanding diabetes [25].

Clinical Manifestations

The clinical manifestations of DN encompass persistent albuminuria, a progressive decline in the GFR, and elevated arterial blood pressure [26]. Patients may present with symptoms such as foamy urine, unexplained proteinuria, manifestations of DR, fatigue, foot edema, and hypertension [26]. It is noteworthy that a majority of DN cases are characterized by the presence of proteinuria and hypertension, both of which exacerbate disease progression. Timely detection through urine testing for albumin is paramount in identifying kidney damage at an early stage [26]. Treatment strategies primarily revolve around the management of diabetes and hypertension, with medications, such as angiotensin-converting enzyme (ACE) inhibitors and angiotensin receptor blockers (ARBs), utilized to regulate blood sugar and blood pressure levels [26]. Regular outpatient follow-up is imperative for the successful management of DN [21]. Recognizing the significance of early intervention, prompt treatment is vital in forestalling or delaying the onset of DN or DKD [21].

Diagnostic Criteria

Several diagnostic markers aid in the identification and assessment of DN. Firstly, elevated albumin levels in the urine serve as a crucial indicator, assessed through urinary albumin/creatinine ratio (ACR) or 24-hour urine collection. Microalbuminuria, defined by an ACR falling within the 30-300 mg/g range, signifies early-stage kidney damage, while macroalbuminuria, with an ACR exceeding 300 mg/g, denotes more advanced nephropathy [27]. Additionally, a low GFR, inferred from blood creatinine levels, indicates kidney dysfunction associated with DN [28]. Imaging modalities, including X-rays, ultrasound, CT, and MRI scans, facilitate visualization of the kidneys, enabling the detection of structural abnormalities or alterations in blood flow associated with DN [29]. In select cases where further insight is warranted, a renal biopsy may be conducted to obtain kidney tissue samples for detailed examination, aiding in the confirmation of DN diagnosis and exclusion of other potential causes of kidney damage [30]. Moreover, clinical symptoms, such as peripheral edema, hypertension, and diminished kidney function, may further suggest the presence of DN [31].

Management Strategies

The management of DN entails several critical strategies aimed at addressing its underlying causes and complications.

Treatment of diabetes and high blood pressure: The initial step in managing DN involves effectively controlling diabetes and high blood pressure. This is typically achieved through lifestyle modifications, dietary adjustments, and medications such as ACE inhibitors and ARBs [32].

Blood sugar control: Controlling high blood sugar levels is paramount in individuals with DN. Medications like metformin, glucagon-like peptide-1 (GLP-1) receptor agonists, and sodium-glucose cotransporter-2 (SGLT2) inhibitors are commonly employed [33].

Cholesterol management: Statins are often prescribed to lower cholesterol levels and reduce the amount of protein in urine, thereby assisting in managing kidney damage associated with DN [34].

Kidney scarring: Finerenone, a newer medication, may offer benefits in reducing tissue scarring in DN. It represents a promising intervention in managing the condition by potentially lowering the risk of kidney failure and cardiovascular complications [35].

Advanced stages: In cases of advanced DN or ESKD, treatment options may include a kidney transplant or kidney dialysis to replace lost kidney function [36]. Preventive measures play a crucial role in minimizing the impact of DN. This includes preventing diabetes through lifestyle modifications and early identification of risk factors for DKD [37].

Renal Complications and Impact on Overall Health

CKD can give rise to significant complications that profoundly affect overall health. These complications encompass fluid retention leading to swelling, elevated blood pressure, pulmonary edema, sudden spikes in potassium levels that may impede heart function, anemia, heart disease, weakened bones, heightened risk of erectile dysfunction or diminished fertility, damage to the central nervous system resulting in concentration difficulties or seizures, compromised immune response rendering one more susceptible to infections, pericarditis, and pregnancy-related complications that might necessitate dialysis or kidney transplant for survival [38]. CKD is associated with grave outcomes such as the progression of kidney failure and complications like cardiovascular disease, anemia, and bone disorders [39]. Even mild to moderate renal impairment is correlated with an escalated cardiovascular risk [40]. Preventive measures encompass the maintenance of a healthy weight, abstaining from smoking, effective management of medical conditions, and routine monitoring of kidney function [40]. Timely detection through urine testing for albuminuria is imperative to identify kidney damage at an early stage [41]. Treatment strategies involve reducing cardiovascular risk, regulating blood sugar levels, managing blood pressure, and inhibiting the renin-angiotensin system [41]. Individuals with CKD must receive optimal treatment to mitigate the risk of complications and enhance outcomes [40].

Diabetic neuropathy

Types of Diabetic Neuropathy

Diabetic peripheral neuropathy: This type of neuropathy affects nerves outside the brain and spinal cord, particularly in the feet and hands. It encompasses motor neuropathy, sensory neuropathy, or a combination of both. Regular foot examinations are imperative for individuals afflicted with this form of neuropathy [42,43].

Diabetic sensory neuropathy: This variety entails damage to nerves responsible for conveying touch, temperature, pain, and other sensations from the skin, bones, and muscles to the brain. It may manifest as tingling, numbness, and diminished pain perception [42,43].

Diabetic autonomic neuropathy: This form of neuropathy affects nerves that regulate internal organs such as the heart rate, blood pressure, digestive system, bladder, sexual organs, sweat glands, and eyes. Additionally, it can result in hypoglycemia unawareness [42,44].

**Figure 3:**
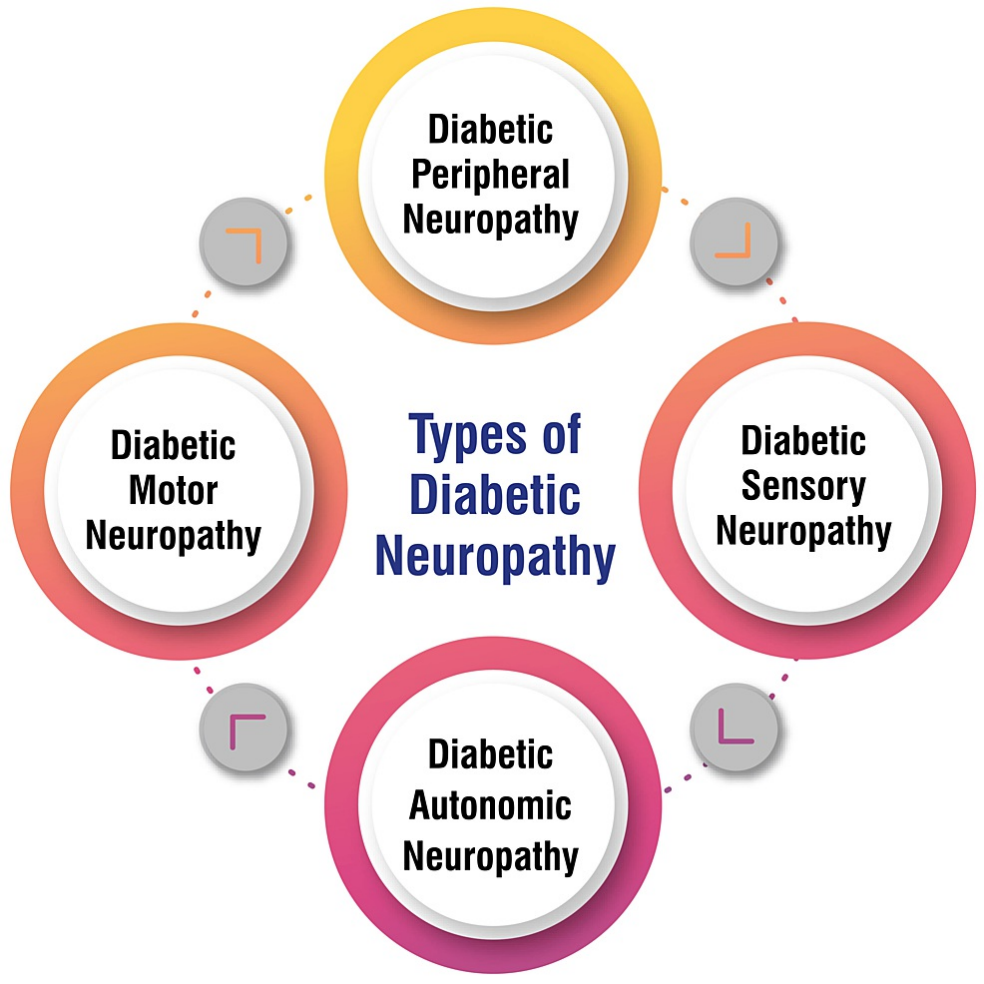
Types of diabetic neuropathy This figure is self-created by the corresponding author.

Diabetic motor neuropathy: This type of neuropathy involves impaired nerves governing muscle movement. It can result in muscle weakness and compromised coordination [43]. Types of diabetic neuropathy are shown in Figure 3.

Pathogenesis

The development of diabetic neuropathy entails the progressive loss of nerve fibers, resulting in a range of clinical manifestations such as pain, paresthesia, and diminished sensation. This loss of nerve fibers follows a pan-modal pattern characterized by a proximo-distal gradient and is linked to microangiopathic changes in peripheral nerve pathology [45,46]. Vascular alterations and the subsequent loss of distal nerve fibers, particularly in small-caliber nerves at epidermal sites, can commence even in individuals with impaired glucose tolerance before affecting nerve fibers in the lower extremities [45]. The mechanism underlying diabetic neuropathy is intricate and involves metabolic cascades instigated by prolonged hyperglycemia. These cascades include heightened polyol pathway flux, the formation of advanced glycation end-products, the release of cytokines, activation of protein kinase C, oxidative stress, and other factors [46]. These metabolic deviations contribute to peripheral nerve damage and predominantly affect the degeneration of distal nerve fibers, rendering the inhibition of individual metabolic factors insufficient for effective treatment. Instead, a combination of inhibitors may offer a promising therapeutic approach to managing diabetic neuropathy [46].

Clinical Manifestations

The clinical manifestations of diabetic neuropathy can vary depending on the specific type of nerve damage present. The most common symptoms are numbness, tingling, burning sensations, aching, cramps, weakness, and pain. These symptoms typically originate in the feet or hands and may progress to affect other areas of the body [42]. Peripheral neuropathy predominantly impacts the feet and legs and is characterized by symptoms such as tingling, numbness, burning, and pain. These symptoms may be alleviated with blood sugar control and appropriate medication [47]. Autonomic neuropathy targets internal organs like the digestive system, blood vessels, urinary tract, and sex organs, resulting in symptoms like bloating, diarrhea, constipation, heartburn, dizziness, low blood pressure, and sexual dysfunction [47]. Proximal neuropathy manifests as pain in the thighs, hips, or buttocks, accompanied by leg weakness [47]. Focal neuropathy can induce sudden pain or weakness in specific nerves, typically affecting areas such as the head or torso [47]. For individuals with diabetes, effectively managing blood sugar levels is paramount to prevent or delay the onset of diabetic neuropathy and its associated complications.

Diagnostic Approaches

Diagnostic approaches for diabetic neuropathy encompass a variety of methods aimed at assessing and diagnosing this condition. Critical laboratory screening tests include hemoglobin A1c and fasting plasma glucose, providing valuable insights into recent diabetes control [48]. Electrophysiologic testing, such as nerve conduction testing and needle EMG, is highly recommended by multiple consensus panels for evaluating diabetic neuropathy [48]. Additionally, noninvasive screening tools like the Rydel-Seiffer tuning fork and monofilaments find utility in detecting peripheral neuropathies, particularly in pediatric patients [2]. Skin biopsies and immunohistochemistry serve as newer diagnostic tools used primarily for research purposes, aiding in assessing small nerve fibers in diabetic neuropathies [48]. Quantitative sensory testing (QST) offers another diagnostic avenue by quantifying small and large nerve fiber functions through responses to temperature and vibratory stimuli [49]. However, the sensitivity of QST can be variable due to factors such as patient cooperation, thereby impacting its diagnostic accuracy [49]. Corneal confocal microscopy (CCM) emerges as a non-invasive imaging modality with promising potential as a surrogate marker for assessing sensory C-fibers in diabetic peripheral neuropathy [49]. In clinical practice, the diagnosis of diabetic neuropathy involves a comprehensive approach that includes symptom assessment, clinical bedside testing, QST, and nerve conduction studies [50]. Specialized screening tests, such as monofilament examination, vibration perception tests, tuning fork assessments, and ankle reflex evaluations, are recommended for screening diabetic neuropathy in patients with diabetes [50]. Early diagnosis of diabetic neuropathy assumes critical importance for effective management and the prevention of complications such as foot ulcers or amputations [50].

Treatment Options

The treatment of painful diabetic neuropathy (PDN) adopts a multifaceted approach aimed at impeding progression, alleviating pain, managing complications, and restoring function. Three primary approaches underlie the treatment of PDN: intensive glycemic control and risk factor management, treatments targeting pathogenetic mechanisms, and symptomatic pain management [51]. Clinical guidelines advocate for a range of medications for pain relief in PDN, including antidepressants like amitriptyline and duloxetine, γ-aminobutyric acid analogs such as gabapentin and pregabalin, opioids, and topical agents like capsaicin [43,51]. Moreover, proposed pathogenetic treatments encompass α-lipoic acid, benfotiamine, and aldose-reductase inhibitors [51]. Promising candidates for future PDN therapy include Na_v_1.7 antagonists, N-type calcium channel blockers, nerve growth factor (NGF) antibodies, and angiotensin II type 2 receptor antagonists [51]. Alternative therapies, such as capsaicin cream, alpha-lipoic acid supplements, acetyl-L-carnitine supplements, transcutaneous electrical nerve stimulation (TENS), and acupuncture, may also relieve pain in diabetic neuropathy [52]. Collaborating closely with healthcare providers to tailor treatment approaches to individual needs and symptoms is crucial. Regular monitoring of blood sugar levels, maintaining a healthy lifestyle, and seeking support from healthcare providers or support groups all play pivotal roles in effectively managing diabetic neuropathy [52].

Impact on Daily Functioning and Quality of Life

Diabetic neuropathy can exert a profound impact on daily functioning and overall QoL. Research indicates that individuals afflicted with diabetic neuropathy may encounter challenges in physical performance, exhibit deficits in postural balance, experience sensorial limitations, and report diminished QoL in comparison to those without neuropathy [53]. Furthermore, diabetic neuropathy can precipitate postural instability, heighten the risk of falling, and impede the execution of activities of daily living [54]. The presence of diabetic peripheral neuropathic pain has been shown to exert a negative influence on health-related QoL, particularly affecting domains associated with physical roles and mental health [55]. Moreover, diabetic polyneuropathy, irrespective of whether it manifests as painful or non-painful, significantly compromises the QoL among individuals with diabetes [56]. Specifically, painful diabetic peripheral neuropathy correlates with impairments in both physical and emotional functioning, symptoms indicative of anxiety and depression, as well as disturbances in sleep patterns [57]. Therefore, the effective management of diabetic neuropathy assumes paramount importance in enhancing the QoL for individuals with diabetes and forestalling further complications associated with this condition.

Interconnections between diabetic retinopathy, nephropathy, and neuropathy

Shared Risk Factors

Shared risk factors for microvascular complications in diabetes, encompassing retinopathy, nephropathy, and neuropathy, are influenced by various factors. Research indicates that the age at the onset of diabetes plays a pivotal role in the development of these complications, with a younger age at the onset correlating with a heightened likelihood of experiencing multiple microvascular complications. Furthermore, gender, elevated body mass index (BMI), dyslipidemia (including total cholesterol, high-density lipoproteins, and low-density lipoproteins), and smoking are implicated as risk factors for DR. Additionally, a longer duration of diabetes, younger age at onset, and the utilization of insulin have been identified as robust predictors of DR. Notably, in Saudi Arabia, the presence of neuropathy and nephropathy emerged as significant risk factors for DR in patients with type 2 diabetes. These findings underscore the critical importance of effectively managing these shared risk factors to mitigate or postpone the onset and progression of microvascular complications in diabetes [58,59].

Common Pathophysiological Mechanisms

The pathophysiological mechanisms underlying the interconnected complications of DR, nephropathy, and neuropathy in diabetes mellitus involve a shared process of microvascular damage triggered by chronic hyperglycemia. This prolonged exposure to high blood sugar levels leads to tissue vulnerability to oxidative stress, inflammation, and endothelial dysfunction, ultimately contributing to structural and functional abnormalities in the microvasculature [60]. Target tissues, such as the retina, kidney, and peripheral nerves, exhibit a heightened susceptibility to intracellular hyperglycemia toxicity due to the distribution of glucose transporters. Poorly controlled blood glucose levels exacerbate this vulnerability, increasing the risk of microangiopathy and subsequent complications [61].

DR, a prevalent microvascular complication in diabetes, stems from vision-threatening damage to the retina induced by hyperglycemia-mediated effects on the retinal microvasculature. This includes basement membrane thickening, capillary permeability alterations, MAs formation, and intravascular coagulation, culminating in retinal ischemia and neovascularization. DR can progress from non-proliferative to proliferative forms, with or without macular edema, significantly impacting vision [62]. Additionally, DN and neuropathy represent significant complications of diabetes. DN is characterized by structural and functional abnormalities in the kidneys resulting from chronic hyperglycemia. Timely detection through microalbuminuria testing is crucial for initiating prompt intervention. Tight glycemic control, aggressive blood pressure management, and cholesterol reduction are pivotal in attenuating disease progression in nephropathy and retinopathy [60]. The pathophysiological mechanisms of DR, nephropathy, and neuropathy involve chronic hyperglycemia-induced microvascular damage, leading to structural and functional aberrations in target organs like the retina, kidneys, and nerves. Early detection and comprehensive management strategies are imperative for mitigating the progression of these complications in diabetic patients.

Bidirectional Relationships and Causality

The relationship between depression and DN appears to be bidirectional, as evidenced by a meta-analysis examining this association. The study revealed that DN may predict the onset of depression, with a pooled odds ratio of 1.46. At the same time, depression may also serve as an indicator of DN, with an odds ratio of 1.22 [63]. This bidirectional link suggests that both conditions can mutually influence each other, emphasizing the necessity for further research to elucidate the underlying mechanisms and devise interventions for this comorbidity. Furthermore, investigations have delved into the interconnections among nephropathy, retinopathy, and autonomic neuropathy in individuals with type 1 diabetes. These conditions are viewed as manifestations of a generalized diabetic microangiopathic process, indicating a common underlying mechanism in diabetes progression [23]. Understanding these relationships is imperative for comprehensive diabetes management and underscores the significance of addressing these interconnected complications collectively. Additionally, a Danish nationwide registry-based cohort study sought to explore the bidirectional associations between DR and significant depression. The findings revealed that while significant depression did not predict the five-year incidence of DR, the utilization of insulin was strongly correlated with incident major depression in patients with type 2 diabetes [64]. This underscores the intricate interactions among psychological factors, disease progression, and treatment modalities in individuals with diabetes.

Screening and management strategies

Importance of Early Detection and Integrated Management

Early detection and integrated management are pivotal in effectively managing DR, nephropathy, and neuropathy in individuals with diabetes. Timely identification of DR is critical for averting vision loss and initiating prompt treatment. Screening programs are crucial in identifying referable DR, characterized by moderate to severe stages of the disease. Adopting artificial intelligence (AI) technologies, such as deep learning algorithms, has markedly improved the automated detection of DR, enhancing the efficiency and accuracy of screening processes [65,66]. For DN, early detection through screening methods like urinary ACR is imperative to intervene before the progression to overt nephropathy. Strategies such as optimizing glycemic control and utilizing treatments like ACE inhibitors or angiotensin II receptor blockers are recommended to manage DN effectively [67,68].

In the case of diabetic neuropathy, lifestyle modifications and mitigating cardiometabolic risk factors are essential for reducing the risk of disease progression. Intensive glycemic control has demonstrated efficacy in preventing neuropathic complications, underscoring the importance of regular screening for neuropathy to manage this complication efficiently [69]. Integrating early detection strategies, such as AI-based screening methods, with comprehensive management approaches tailored to the severity of each complication is crucial in preventing the progression of DR, nephropathy, and neuropathy in individuals with diabetes. Regular screenings, optimized glycemic control, and timely interventions are critical components of an integrated approach to managing these diabetes-related complications effectively [70].

Screening Guidelines for Diabetic Complications

Screening guidelines for diabetic complications advocate universal screening for prediabetes and diabetes among all adults. The American Diabetes Association recommends screening individuals aged 45 years or older, irrespective of risk factors, using fasting plasma glucose levels, two-hour plasma glucose levels during an oral glucose tolerance test, or HbA1c levels. Additionally, adults who are overweight or obese and have one or more risk factors should also undergo screening, with repeat screenings recommended at least every three years if results are normal [71]. Regarding DR, screening should be conducted using validated approaches and methodologies. Individuals with type 1 or type 2 diabetes should undergo regular screenings, with prompt referral to an ophthalmologist upon detection of DR. The frequency of subsequent examinations is contingent on the individual's risk factors and the progression of retinopathy [67]. For DN, optimizing glycemic control is paramount for prevention. Screening methods, such as the urinary ACR, are recommended to detect early signs of diabetic renal disease. Treatment with ACE inhibitors or angiotensin II receptor blockers is advised for hypertensive patients exhibiting microalbuminuria or albuminuria [72]. In the case of diabetic neuropathy, lifestyle modifications and minimizing cardiometabolic risk factors are imperative. Regular screening is advocated to effectively manage this complication, with intensive glycemic control playing a pivotal role in preventing neuropathic complications of diabetes [66]. These screening guidelines underscore the significance of early detection, timely intervention, and optimized control of blood glucose levels to prevent the progression of diabetic complications, such as retinopathy, nephropathy, and neuropathy, in individuals with diabetes.

Multidisciplinary Approach to Patient Care

A multidisciplinary approach to patient care involves a collaborative effort among healthcare professionals from diverse medical disciplines to address the multifaceted needs of patients. This coordinated approach in healthcare entails doctors, nurses, dieticians, and other medical staff members working together to develop treatment plans and deliver high-quality care [73-75]. The benefits of multidisciplinary patient care are manifold, including improved healthcare services, enhanced access to facilities and information, heightened satisfaction, early diagnosis and treatment, high-quality clinical care, and psycho-social support. For healthcare professionals, this approach offers advantages such as improved care coordination, enhanced patient outcomes, reduced duplication of services, streamlined treatment pathways, and increased opportunities for collaboration among health experts [73-75]. Multidisciplinary care teams are pivotal in managing complex or chronic health conditions like cancer. These teams conduct comprehensive assessments of patients' situations, promote health initiatives, provide community education, and devise patient-centered treatment plans through collaborative efforts. By effectively collaborating, multidisciplinary teams mitigate medical errors, enhance patient safety, improve outcomes, shorten hospital stays, and decrease unplanned hospital readmissions [75-77].

Lifestyle Interventions and Pharmacotherapy

Lifestyle interventions and pharmacotherapy are essential components in preventing and managing DR. Lifestyle modifications encompass weight reduction, healthy dietary habits, and engaging in physical activity, which have effectively reduced blood pressure and serum lipids and improved glycemic control [78]. A study conducted in Finland revealed that an intensive lifestyle intervention over four years among overweight and obese individuals with impaired glucose tolerance decreased the risk of retinal MAs and lowered serum triglyceride levels [78]. Similarly, another study suggested that physical activity could reduce the risk of DR progression by 40% [79]. Pharmacotherapy options involve medications aimed at controlling blood sugar levels, such as insulin or metformin, regulating blood pressure with ACE inhibitors, and managing cholesterol with statins [11]. Regular screening and monitoring of blood sugar, blood pressure, and cholesterol levels are imperative in preventing DR [80]. Furthermore, maintaining a healthy weight, adhering to a balanced diet, and engaging in regular physical activity significantly reduce the risk of DR and its associated complications [80].

Patient Education and Self-Management

Patient education and self-management are fundamental pillars in effectively managing DR. The research underscores the pivotal role of patient education in inspiring proactive behaviors and fostering self-management practices for DR [11]. Strategies to empower patients include online educational initiatives designed to augment knowledge, competence, and adherence to DR care protocols [11]. Healthcare providers play a critical role in promoting eye health among individuals with diabetes by ensuring patients comprehend the ramifications of diabetes on ocular health, advocating for regular eye examinations, and endorsing self-care practices such as blood sugar regulation, healthy lifestyle choices, and medication adherence [81]. Adopting coping strategies for coping with DR entails educating oneself about the condition, scheduling routine comprehensive eye exams, managing blood sugar levels, controlling blood pressure and cholesterol, adhering to medication regimens, implementing lifestyle modifications, utilizing visual aids, seeking emotional support, participating in support groups, and accessing rehabilitation services as needed [81]. Self-management encompasses routine blood pressure monitoring, discussing pregnancy plans with healthcare providers for potential treatment adjustments, adhering to a nutritious diet, engaging in regular physical activity, abstaining from smoking, and adhering to prescribed medication regimens [82]. These self-care practices profoundly influence treatment outcomes and the progression of DR.

Future directions and challenges

Emerging Research Areas

**Figure 4:**
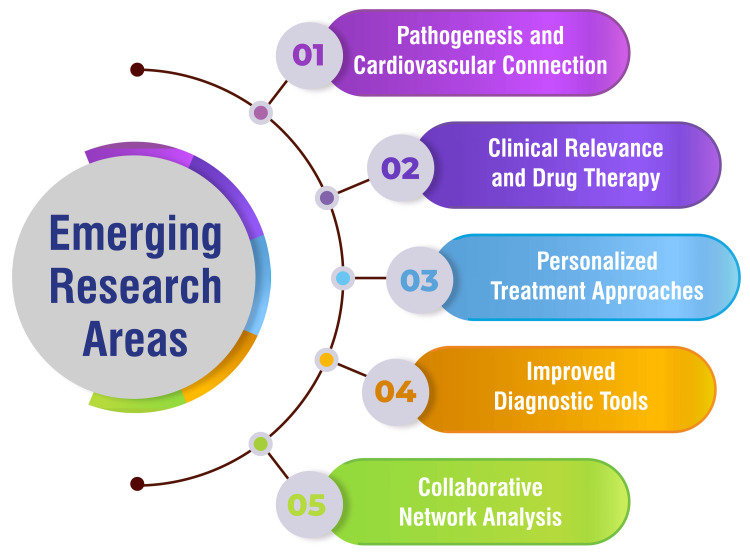
Emerging research areas This figure is self-created by the corresponding author.

Research is currently focused on unraveling the pathogenesis of DN and DR and elucidating their connection with cardiovascular health. This inquiry unveils the underlying mechanisms linking these microvascular complications to cardiovascular outcomes [83]. Future investigations are anticipated to delve into the clinical significance of DN and DR, with particular attention to drug therapy. Exploring novel treatment modalities and pharmaceutical interventions for DR and nephropathy is imperative for enhancing patient outcomes and effectively managing these complications [83]. There is a burgeoning interest in developing personalized treatment approaches for DR, nephropathy, and neuropathy. Customizing treatments based on individual patient characteristics, risk factors, and disease progression holds promise for more efficacious management of these complications [84]. Advancements in diagnostic tools for the early detection of DR, nephropathy, and neuropathy are a focal point of current research efforts. Progress in diagnostic technologies can facilitate the identification of these complications at their nascent stages, enabling timely interventions and improved patient outcomes [84]. Studies are also exploring collaborative network analyses to identify key stakeholders in the research landscape of DN and DR. Gaining insights into the most influential countries, institutions, and authors can foster collaborations and propel advancements in knowledge within this domain [83]. Emerging research areas are shown in Figure 4.

Novel Therapeutic Approaches

**Figure 5:**
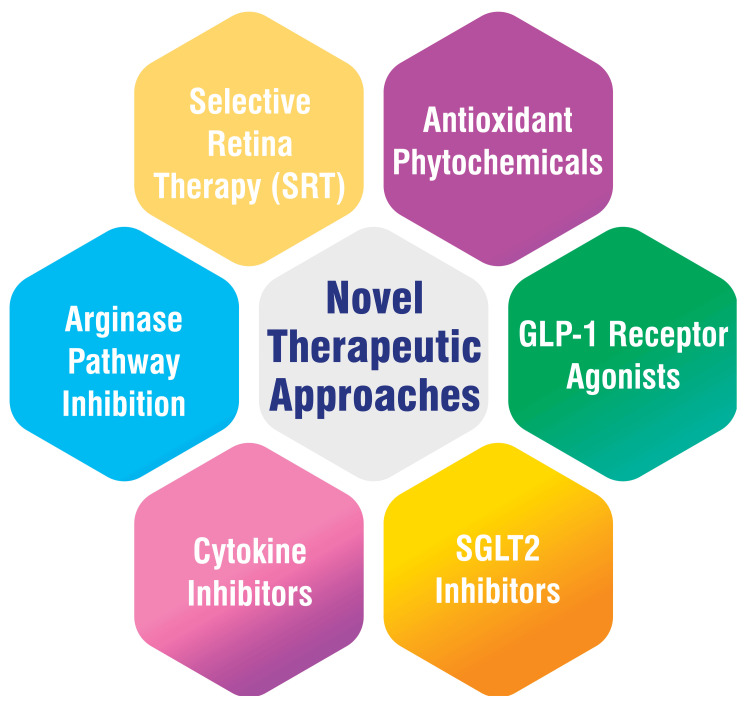
Novel therapeutic approaches This figure is self-created by the corresponding author.

Various therapeutic approaches are being explored to address the complexities of diabetic complications, including using antioxidant phytochemicals to mitigate oxidative stress and enhance antioxidant status through pathways such as silent information regulator T (SIRT1) and Forkhead box O (FOXO), along with micro RNAs [85]. GLP-1 receptor agonists are being investigated for their ability to improve glucose control, with emerging evidence suggesting additional benefits beyond glycemic management, including a potential reduction in the risk of cardiovascular events [86]. SGLT2 inhibitors represent another pharmacological avenue, aiding in reducing blood glucose levels by promoting urinary glucose excretion. These agents have shown promise in mitigating cardiovascular risks, kidney complications, and amputations associated with diabetes [86]. Targeting inflammatory cytokines implicated in the pathogenesis of DR is being explored as a potential therapeutic strategy to impede disease progression [87]. Similarly, inhibiting adhesion molecules involved in inflammatory cell attachment to blood vessels may alleviate inflammation and enhance retinal blood flow [87]. The inhibition of the arginase pathway, which contributes to nitric oxide production involved in DR development, can slow disease progression [87]. Selective retina therapy (SRT) has emerged as a novel treatment approach for clinically significant DME. This method employs a laser to selectively target the retina, potentially enhancing visual acuity and reducing reliance on injections [87]. Novel therapeutic approaches are shown in Figure 5.

## Conclusions

In conclusion, this comprehensive review has illuminated the intricate relationship between DR, nephropathy, and neuropathy. It offers valuable insights into their shared pathophysiological mechanisms, bidirectional relationships, and profound impact on patient health and well-being. It underscores the imperative for healthcare professionals to adopt a holistic approach to diabetes management, integrating screening, early intervention, and multidisciplinary care to mitigate the progression of complications. Furthermore, the review highlights the pressing need for continued research and collaboration to elucidate the underlying mechanisms, identify novel therapeutic targets, and assess the efficacy of integrated care models in preventing or delaying the onset and progression of diabetic complications. By fostering interdisciplinary collaboration and knowledge exchange, future research endeavors promise to improve outcomes and QoL for individuals with diabetes.
